# To Compare PubMed Clinical Queries and UpToDate in Teaching Information Mastery to Clinical Residents: A Crossover Randomized Controlled Trial

**DOI:** 10.1371/journal.pone.0023487

**Published:** 2011-08-12

**Authors:** Ladan Sayyah Ensan, Masoomeh Faghankhani, Anna Javanbakht, Seyed-Foad Ahmadi, Hamid Reza Baradaran

**Affiliations:** Center for Educational Research in Medical Sciences, Medical Education and Development Center, Tehran University of Medical Sciences, Tehran, Iran; Children's Hospital of Eastern Ontario, Canada

## Abstract

**Purpose:**

To compare PubMed Clinical Queries and UpToDate regarding the amount and speed of information retrieval and users' satisfaction.

**Method:**

A cross-over randomized trial was conducted in February 2009 in Tehran University of Medical Sciences that included 44 year-one or two residents who participated in an information mastery workshop. A one-hour lecture on the principles of information mastery was organized followed by self learning slide shows before using each database. Subsequently, participants were randomly assigned to answer 2 clinical scenarios using either UpToDate or PubMed Clinical Queries then crossed to use the other database to answer 2 different clinical scenarios. The proportion of relevantly answered clinical scenarios, time to answer retrieval, and users' satisfaction were measured in each database.

**Results:**

Based on intention-to-treat analysis, participants retrieved the answer of 67 (76%) questions using UpToDate and 38 (43%) questions using PubMed Clinical Queries (P<0.001). The median time to answer retrieval was 17 min (95% CI: 16 to 18) using UpToDate compared to 29 min (95% CI: 26 to 32) using PubMed Clinical Queries (P<0.001). The satisfaction with the accuracy of retrieved answers, interaction with UpToDate and also overall satisfaction were higher among UpToDate users compared to PubMed Clinical Queries users (P<0.001).

**Conclusions:**

For first time users, using UpToDate compared to Pubmed Clinical Querries can lead to not only a higher proportion of relevant answer retrieval within a shorter time, but also a higher users' satisfaction. So, addition of tutoring pre-appraised sources such as UpToDate to the information mastery curricula seems to be highly efficient.

## Introduction

With increasing medical literature, learning information management is crucial for clinicians to make them competent to find the best evidence in a short time [1]. In this context the important issue for clinicians is identifying sources which can provide them with reliable, relevant and readable information [2].

Many evidence based medicine workshops and courses have been conducted all over the world to teach clinicians and medical students information management. Most of them focus on principals of searching in resources such as PubMed and especially PubMed Clinical Queries [3]–[4], which is not available bedside and users also need critical appraisal skill to decide on applying retrieved information into daily practice. Whilst some other workshops focus on 5S model as a reliable and optimum approach in order to seek for evidence-based information in systems, summaries, synopses, syntheses and studies arranged through the highest to the lowest level resources, respectively [5]–[6]. However, recently “6S” model is introduced (systems, summaries, synopses of syntheses, syntheses, synopses of studies, and studies) [7]. Both models suggest looking for the needed information at the highest level and proceeding to lower levels in case of failure to find the relevant evidence [6]. Therefore, it seems that learning search within the higher level resources is at least as important as learning search within lower level resources since it may change inefficient information-seeking behavior of physicians [2]. Some studies have compared different medical information resources to suggest the best resources fulfilling trainees' need in practice. Although some of them have compared searching PubMed with UpToDate [8], and searching MEDLINE prior to pre-appraised sources with the reverse protocol [9] it remains unclear which information source should be more emphasized in evidence based medicine workshops.

Since a) computerized decision support systems are not well developed yet, b) using Clinical Queries is reported to facilitate timely retrieval of results in MEDLINE [10], c) UpToDate is reported to be the best “summary” source in the previous studies [11]–[13] the investigators of this study aimed to compare the proportion of relevantly answered clinical questions, time spent to find the answers, and users' satisfaction using PubMed Clinical Queries and UpToDate during a workshop.

## Methods

### Participants and Setting

After obtaining the ethical approval from Medical Education and Development Centre (MEDC) affiliated to Tehran University of Medical Sciences (TUMS), this cross-over randomized trial was conducted in February 2009 at TUMS. MEDC ethics committee agreed with verbal consent. Participants were postgraduate year-one or two residents at TUMS studying in 10 different residency programs including cardiology, pediatrics, emergency medicine, psychiatry, pathology, anesthesiology, radiology, obstetrics and gynecology, internal medicine and urology. They were recruited to participate in a one-day information mastery workshop. The Investigators explained design and purpose of the study to participants and verbal consent was obtained as well.

### Interventions

Through a one-hour lecture, participants were taught principles of Information Mastery including “5S” approach to information resources. (Table 1) The consented participants were randomly assigned to two groups with equal size using UpToDate or PubMed Clinical Queries as the first resource, they were then asked to repeat the exercise using the alternative. In each database they were asked to answer 2 clinical questions. Questions were randomly assigned to participants in a way each participant received a question of diagnosis and a question of therapy, No one search similar questions using two databases, and all questions were also searched in both resources. Before beginning to search, each participant used a self-learning slide-show in power point format demonstrating the instruction on how to use the resource. (Table 1) Then they were given 10 minutes to get familiar with it.

**Table 1:** The detailed content of lecture and self-learning PowerPoint slid-show.

The content of Lecture	
1	Importance of Information mastery and the clinicians' need to it
2	Introduction of 5s or 6s pyramid and how to use it
3	Introduction the 20 resources and classification of them to predigested and raw databases
4	Explaining the characteristics of predigested and raw databases
5	Familiarization with search strategy of 10 databases

The content of self-learning powerpoint slide-shows	
1	How to ask a formulated and answerable question (PICO)
2	How to choose a suitable keyword according to our database and our question
3	Introduction of different parts of UpToDate and PubMed Clinical Queries and their benefits
4	Demonstrating how to search in UpToDate and PubMed Clinical Queries effectively

16 clinical scenarios, with definite answers, followed by a formulated question in the PICO (Patient, Intervention, Comparison, Outcomes) format were selected from the website of the Center of Evidence Based Medicine of the University of Toronto. [14]. The questions were focused on eight clinical fields including child health, critical care, gastroenterology, general practice, general surgery, geriatrics, neonatology and physiotherapy. From each field one question of diagnosis and one question of therapy were selected. Software designed by Microsoft Excel Visual Basic for Application was used to provide participants with questions.

**Figure 1:**
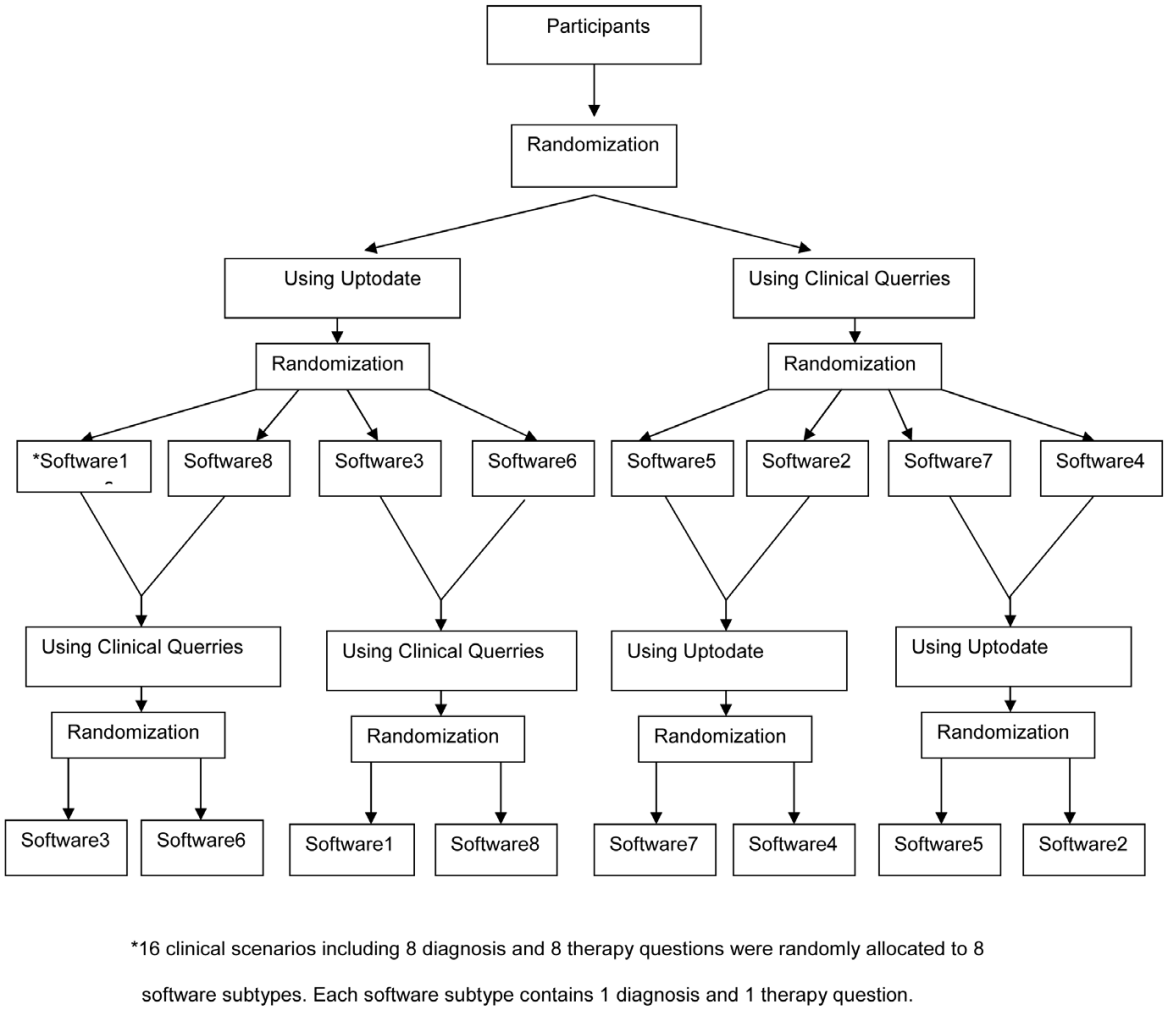
Question randomization flow diagram.

Randomization sequence was generated by Random Allocation Software version1.0.0 using simple random method. Sequentially numbered sealed opaque envelops were used to conceal the allocation. Each participant received one envelope containing the randomization code (Figure 1). Each code indicated the first allocated resource followed by the number of randomly assigned software subtype, the second resource, and its randomly allocated software subtype (ie: U3CQ8). They were not allowed to open the envelope until everyone had his own. Blinding was not applicable to the users and outcome assessors because they could recognize the layout of the resources.

### Measurements

The primary outcome measures of the study were: a) answer retrieval, and b) time to answer retrieval. The secondary outcome measures were: a) user satisfaction, and b) user interaction with PubMed Clinical Queries.

Participants' baseline characteristics including age, gender, type and the year of specialty or subspecialty, and also prior use of allocated resources were recorded using a checklist. Basic computer skills and prior familiarity with resources were measured by a five-point Likert scale.

The answers and time to retrieve them was also saved by the software. Because of time limitation of the workshop and the importance of time-effective answer retrieval in bedside, the software assigned maximum 20 minutes to each scenario to be answered. If participants had asked for more time they would have been provided with it. They were also able to stop the program whenever they found the answer and the software was able to calculate the time. Finally, investigators assessed the relevancy of retrieved information by participants to the answer mentioned in the website of the Center of Evidence Based Medicine of the University of Toronto and they also checked if the layout of saved information is compatible with the layout of the information source using by participant [14].

The measures of users' satisfaction including interaction with the resource, amount and accuracy of the retrieved information, and overall satisfaction were recorded using a questionnaire [12]. The measures of user interaction with PubMed Clinical Queries were also recorded using a self-administered checklist [Table 2].

**Table 2:** Users' interaction factors with PubMed Clinical Queries.

Did you start Searching by Clinical Study Category or Find Systematic Reviews?
Did you find the answer by searching Clinical Study Category or Find Systematic Reviews?
Did you find the sufficient answer of your question in abstract or full text?
Which criteria did you consider to select the article answering your question?

### Statistical Analysis

In this study proportion of retrieved answer, time to answer retrieval, and the measure of users' satisfaction were compared by the McNemar test, Log Rank survival analysis, and Wilcoxon test respectively. Each analysis was performed on all data, questions of diagnosis, and questions of therapy.

In order to do intention-to-treat analysis we assigned the outcomes to the resource which they were basically allocated to use via the randomization sequence. Whenever there was a failure to record the answer or time to answer (mostly due to technical errors), data imputation was used to substitute the missing values. These substituted values were calculated based on other participants' outcomes. Finally, results of intention-to-treat and per-protocol analysis were compared using sensitivity analysis. SPSS V.16 was used for the whole process of analysis and a P<0.05 was considered significant.

## Results

### Characteristics of the participants

**Figure 2:**
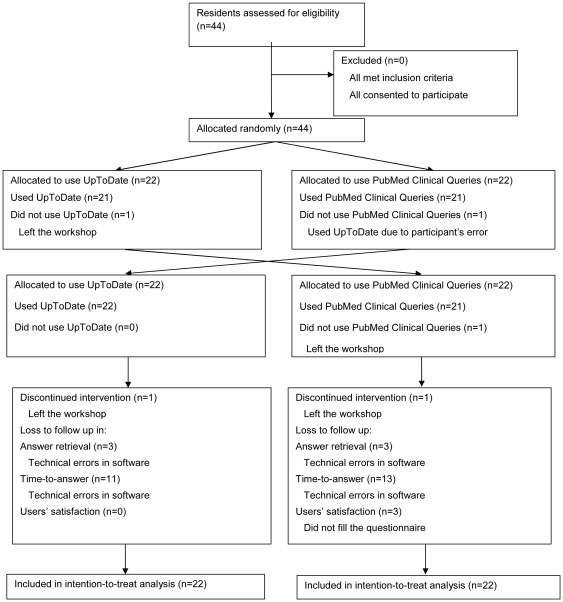
Participants flow diagram.

Forty four participants were recruited to the study [Figure 2]. Twenty six (63%) were male. Thirty seven (90%) were in the first year of the residency program. The mean age of participants was 32 years (SD = 3). The median of their basic computer skills was medium (3 out of 5 in a five-point Likert scale).

Baseline characteristics including prior use of and familiarity with the two resources were comparable between the two groups.

### Answer retrieval

Participants retrieved relevant answers to 67 (76%) questions using UpToDate compared to 38 (43%) questions using PubMed Clinical Queries (P<0.001).

The answer to the questions of diagnosis was retrieved 38 (86%) by UpToDate users compared to 25 (57%) by PubMed Clinical Queries users (P = 0.004).

**Figure 3:**
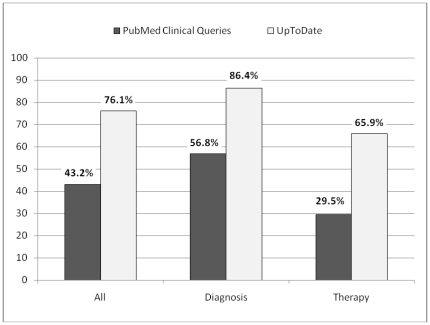
Comparison of answer retrieval in PubMed Clinical Queries and UpToDate. The percentage of whole answered questions is compared by the columns on the left, while the percentage of answered questions of diagnosis and therapy are compared by the columns on middle and on the right, subsequently.

For questions of therapy, UpToDate users answered 29 (66%) of questions compared to 13 (29%) of questions answered by PubMed Clinical Queries users (P = 0.002) [Figure 3].

### Time to answer retrieval

Survival analysis showed that median time to answer retrieval was 17 min (95% CI: 16 to 18) among UpToDate users compared to 29 min (95% CI: 26 to 32) among Pubmed Clinical Queries users (P<0.001).

The median time to answer retrieval for the questions of diagnosis was estimated to be 16 min (95% CI: 15 to 16) using UpToDate versus 25 min (95% CI: 21 to 29) using PubMed Clinical Queries (P<0.001).

For questions on therapy the median time to answer retrieval was 18 min (95% CI: 16 to 20) for UpToDate users and 43 min (95% CI: 42 to 43) for PubMed Clinical Queries users (P = 0.011).

### Users' satisfaction

Results of the users' satisfaction survey are summarized in Table 3. Users were satisfied with accuracy of retrieved answers from UpToDate significantly more than PubMed Clinical Queries .They also reported significantly easier interaction with UpToDate compared to the PubMed Clinical Queries. Similarly, Overall satisfaction was higher among UpToDate users.

**Table 3:** Comparision of users' satisfaction in Pub Med Clinical Queries and UpToDate.

	UpToDate	PubMed Clinical Querries	P
Interacting with system*, median (IQR)	4 (3 to 4)	2 (2 to 3)	<0.001
Amount of retrieved information*, median (IQR)	3 (2 to 4)	3 (3 to 4)	0.114
Accuracy of content*, median (IQR)	3 (2 to 3.75)	2 (1 to 3)	<0.001
Overall satisfaction*, median (IQR)	3 (3 to 3.75)	2 (1 to 2.75)	<0.001

*Measured by five points Likert scales; “1” refers to the lowest satisfaction level and “5” refers to the highest.

IQR: Interquartile range.

### User interaction with PubMed Clinical Queries

PubMed Clinical Queries users reported that they started searching 46 (65%) out of 88 questions in “Clinical Study Category” box and 25 (35%) questions in “Find Systematic Review” box.

Out of 34 answered questions, the users found the answer of 24 (83%) in the “Clinical Study Category” box compared to 5 (17%) in the “Find Systematic Review” box.

The abstract of the articles were used in 24 (77%) out of 34 retrieved answers in PubMed Clinical Queries and users did not need full text to find the answers.

Relevancy was the most frequent criterion to select the article for 24 (77%) out of 34 retrieved answers.

### Sensitivity analysis

Per- protocol analysis showed an answer retrieval rate of 74% in UpToDate compared to 41% in PubMed Clinical Queries (P<0.001).

In addition, per-protocol survival analysis estimated a median time to answer retrieval of 15 min for UpToDate compared to 30 min for PubMed Clinical Queries (P<0.001).

Per-protocol comparison of satisfaction factors between UpToDate and PubMed Clinical Queries showed a significant difference regarding the interaction with database (P<0.001), accuracy of content (P = 0.001) and overall satisfaction (P<0.001).

Comparing the results of per-protocol and intention-to-treat analyses showed that no test yielded a different result and also the outcomes were similar.

## Discussion

The results of this study indicated that first time users using UpToDate could answer a higher proportion of questions within a shorter time rather than Pub Med Clinical Queries. In addition, UpToDate users reported a higher satisfaction regarding interaction with system, accuracy of the content and also overall satisfaction.

In a previous study, Patel and colleagues showed that when searching MEDLINE preceded pre-appraised sources (including UpToDate, ACP Journal Club and Cochrane Library), most of the questions (80%) were answered with MEDLINE and little further questions (5%) with the pre-appraised sources; while using the reverse search protocol, a lower proportion of questions (64%) were answered with pre-appraised sources and a considerable proportion of questions (23%) with MEDLINE. In contrast, considering the time factor, a higher proportion of questions were answered in less than 5 minutes when pre-appraised sources were searched prior to MEDLINE (26% vs. 55%) [9]. These results could show that the content coverage of MEDLINE is more comprehensive; but in limited time, pre-appraised sources are more rewarding. In another study, Hoogendam and colleagues reported a higher answer retrieval rate for UpToDate compared to Pub Med (83% vs. 63%) and also a shorter time to answer retrieval (241 vs. 291 seconds) [8]. Similarly, Thiele and colleagues showed that not only users of UpToDate were more likely than users of PubMed to answer the questions correctly but also UpToDate were faster than PubMed in answer retrieval. Indeed, subjects had the most confidence in UpToDate [15]. Most of the results of these studies support our findings. However, in both of these studies Clinical Queries was not emphasized in searching MEDLINE. While Demner-Fushman and colleagues showed that using Clinical Queries facilitates timely retrieval of results in MEDLINE [10], not focusing on Clinical Queries might be the reason of the low timely retrieval rate in MEDLINE in those studies.

PubMed Cilnical Queries is a set of search filters for separating valid and relevant articles out of the repository of PubMed citations. Thus limits its clinical efficiency; because: a) Searching for one question may yield multiple high quality articles that present different answers, which the clinician does not have time to evaluate comprehensively. b) Few articles compare all management options for a given health problem. Therefore if the clinicians intend to decide between all possible options, they would have to review several studies systematically to inform their decision making. This is time consuming and also requires expertise.

On the other hand, UpToDate is highly efficient; because a) the information is organized in entries rather than articles; each discusses a complaint (e.g. chest pain), disease (e.g. acute coronary syndrome) or a category (e.g. diagnosis) of a disease; if a special issue needs further discussion, another entry would be specified to it (e.g. cholesterol lowering after an acute coronary syndrome). Thus, the clinician is guided to alternation and is not overwhelmed with information. b) The information is provided by integrating the best available evidence by experts to address all management options for a given health problem and most of the recommendations are graded on the basis of their level of evidence. Thus, clinicians can use the recommendations knowing that all options are considered and the best one is recommended.

The study limitations include: a) Whilst the native language of the participants was Persian (Farsi), the databases were in English. Thus may increase the time to retrieve answer, b) Unfamiliarity of participants with information management skills and inadequate competency for searching PubMed Clinical Queries compared to UpToDate inspite of equal prior training which might be the reason of such a low answer retrieval in this source, c) limited time for learning, practicing, and also searching for the answer of each question, d) using limited number of questions compared to the previous studies, e) limited questioned clinical categories and failure to include other important categories (e.g. prognosis), and f) Technical problem with the internet speed in the 2nd workshop which leaded to such a long median time to answer retrieval for both databases compared to the similar studies.

However, this study has the following strengths: a) conducting a randomized cross-over rather than self-control trial during the workshop, b) providing training to use both PubMed Clinical Queries and UpToDate by the self-learning slide shows, c) providing participants with clinical scenarios and formulated foreground questions, d) measuring the time to answer retrievals accurately using special designed software, e) verifying all answers for relevance.

Based on the findings of this study, we recommend addition of tutoring pre-appraised resources such as UpToDate in information mastery workshops; because they seem to be more rewarding and faster, so more applicable in the daily practice; furthermore, they can enhance lifetime learning competencies among physicians. This study can be a signal to conduct studies comparing two different EBM workshop curricula regarding participants' satisfaction, effects on clinically important outcomes, medical errors, and costs. The results of such studies may make refinements in EBM workshop curricula.
